# C-Reactive Protein and Gamma-Glutamyltransferase Concentrations in Relation to the Prevalence of Type 2 Diabetes Diagnosed by Glucose or HbA1c Criteria in Chinese Adults in Qingdao, China

**DOI:** 10.1155/2010/761715

**Published:** 2010-11-07

**Authors:** J. Ren, Z. C. Pang, W. G. Gao, H. R. Nan, S. J. Wang, L. Zhang, Q. Qiao

**Affiliations:** ^1^Department of Epidemiology and Health Statistics, Shandong University, Jinan 250012, China; ^2^Department of Non-Communicable Disease Prevention, Qingdao Municipal Centre for Disease Control and Prevention, no. 175, Shandong Road, Qingdao 266071, China; ^3^Department of Public Health, University of Helsinki, 00014 Helsinki, Finland; ^4^Diabetes Unit, Department of Chronic Disease Prevention, National Institute for Health and Welfare, 00014 Helsinki, Finland; ^5^Qingdao Endocrinology and Diabetes Hospital, 266071 Qingdao, China; ^6^Hong Kong Institute of Diabetes and Obesity, Hong Kong, China

## Abstract

*Aims*. To investigate the association of C-reactive protein (CRP) and gamma glutamyltransferase (GGT) concentrations with newly diagnosed diabetes defined by either glucose or HbA1c criteria in Chinese adults. *Methods*. A population-based cross-sectional study was conducted in 2006. Data from 1167 men and 1607 women aged 35–74 years were analyzed. Diabetes was defined according to either glucose or HbA1c criteria alone. *Results*. Compared with nondiabetes, multivariate-adjusted OR (95%CI) was 1.13 (0.90,1.42) in men and 1.21 (1.00,1.45) in women for CRP and 1.42 (1.18,1.72) and 1.57 (1.31,1.87) for GGT, respectively. Neither CRP nor GGT was associated with the presence of diabetes defined by the HbA1c criterion. *Conclusions*. The effect of elevated CRP on diabetes defined by the glucose criterion was mediated through obesity, but elevated GGT was an independent risk factor for diabetes in this Chinese population. None of the two was, however, associated with the elevated HbA1c concentrations.

## 1. Introduction

A new large Chinese national survey [1] is in consistent with previous report from China [2] showing that type 2 diabetes has become a seriously public health threat in China.The studies suggest that China is taking over India and becoming the epicenter of diabetes in the world. In these previous studies, type 2 diabetes has been defined according to fasting (FPG) and/or 2-hour plasma glucose criteria after ingesting 75 g oral glucose load (OGTT) [3]. Recently haemoglobin A1c (HbA1c) test has been adopted as a diagnostic criterion for diabetes [4]. The impact of changes in diagnostic criteria on prevalence of diabetes and differences in phenotypes detected by different criteria are, however, still less known. 

C-reactive protein (CRP) is a nonspecific biomarker of acute inflammation and is produced primarily in the liver. Several prospective studies had shown that serum CRP accelerate or increase the development of diabetes [5–9], particularly in women [8, 9]. There is increasing evidence showing that liver enzymes, such as gamma-glutamyltransferase (GGT), used as a marker of alcohol consumption or liver disease, show a dose-response relation with incident diabetes even within its normal range [10] and may also predict the development of diabetes in both genders independent of traditionally risk factors [11, 12]. 

However, type 2 diabetes in all above-mentioned studies was diagnosed by FPG and/or OGTT not by HbA1c criteria. In this study, the association of CRP or GGT with type 2 diabetes diagnosed by either glucose or HbA1c criteria is investigated based on a cross-sectional population-based Chinese study in Qingdao, China.

## 2. Subjects and Methods

### 2.1. Study Population

A total of 6100 individuals aged 35–74 years who had lived in Qingdao City for at least 5 years were recruited in 2006 with stratified random cluster sampling from 3 urban districts (Shinan, Shibei and Sifang) and 3 rural districts (Huangdao, Jiaonan and Jimo). Among these, 5355 individuals participated in the study, with a response rate of 87.8%. The inclusion criteria for the current study were (1) newly diagnosed diabetes by both FPG and/or 2 hours plasma glucose criteria (2) no data missing for body mass index (BMI), waist circumference (WC), blood pressure measurements, CRP, GGT, and HbA1c. A total of 2774 (1167 men) subjects with full required information were included.

 Height and weight were measured with participants wearing light clothes and without shoes, and BMI was calculated by dividing weight (Kg) by height (m) squared. WC was measured at the middle point between the rib cage and top of the iliac crest to the nearest 0.1 cm. Three consecutive blood pressure readings, apart by at least 30 seconds, were taken from the right arm of seated subjects, and the average of the three readings was used in the data analysis. Smoking status was classified as current smokers (smoking daily regardless of the amount and type of smoking) and nonsmokers (including ex-smoking, smoking now and then and not smoking at all). Alcohol-drinking status was defined as current drinkers (drink frequently regardless of the amount and type of alcohol) and nondrinkers (including ex-drinking, drinking now and then or not drinking at all). Family history of diabetes was defined as having at least one of parents, sibling or offspring with diabetes. School years were divided into two levels (≤9 or >9 school years). Occupational activities were categorized into light (managerial staff), moderate (teacher/doctor/nurse), and heavy (worker or farmer). All subjects without a prior history of diabetes underwent a standard OGTT. Blood samples were transported in a dark box with ice to the laboratory no more than 6 hours after drawing and stored at −80°C. The lab assays were performed in the central laboratory of Qingdao Hiser Medical Center using Olympus AU analyzers (Olympus, Tokyo, Japan). Plasma glucose levels were determined by the glucose oxidase method. Fasting serum triglycerides (TG) and total cholesterol (TC) were determined by enzymatic method while fasting serum CRP and high-density lipoprotein cholesterol (HDL-C) by direct method. Low-density lipoprotein cholesterol (LDL-C) was calculated using the Friedewald equation. GGT and alanine aminotransferase (ALT) were measured by using an International Federation of Clinical Chemistry (IFCC) method. HbA1c was measured using an immunoturbidimetry method (Tina-qu.a A1C HIT 917 large; Roche Diagnostics). The HbA1c concentration was calculated by using the formula provided by Roche Diagnostics: [calculated HbA1c (%) = 0.81 × HbA1c (test result) + 2.39] to match the values with those found in the new IFCC standardization procedure, a conventional high performance liquid chromatography (HPLC) method [13]. The calculated HbA1c was subsequently used in the data analysis. The reference range for the calculated HbA1c was 4.3%–5.8%. Fasting insulin concentration was measured in 2125 individuals using the chemiluminescence immunoassay method (Abbott AxSym). Fatty Liver Index (FLI)-based on GGT, TG, BMI and WC-, was calculated as follows [14]:

FLI = (e0.953∗loge (triglycerides) + 0.139∗BMI + 0.718∗loge (ggt) +0.053∗waist circumference − 15.745)/(1 + e0.953∗loge (triglycerides) + 0.139∗BMI + 0.718∗loge (ggt) + 0.053∗waist circumference − 15.745)∗100. 

An FLI ≤ 30 rules out and an FLI ≥ 60 indicates hepatic steatosis as detected by ultrasoundgraphy.

The Ethics Committee of Qingdao Municipal Hospital approved the study. Verbal or written consent was obtained from each participant prior to the data collection.

### 2.2. Classification of Diabetes

Diabetes was defined according to the 2006 World Health Organization (WHO)/International Diabetes Federation (IDF) criteria [3] and International Expert Committee Report on the role of the HbA1c assay for diabetes [4]. Subjects who reported a history of diabetes and who were under treatment with either insulin or oral antidiabetic agents were considered as previously diagnosed diabetes, regardless of their fasting plasma glucose levels. Diagnosed diabetes was excluded from the data analysis to reduce the potential effluence of the anti-diabetic medications and the diabetic complications. Newly diagnosed diabetes was defined if there was an FPG level of ≥7.0 mmol/L and/or 2hPG level of ≥11.1 mmol/L regardless of the HbA1c concentration. Alternatively, newly diagnosed diabetes was determined if the calculated HbA1c ≥6.5% in spite of the glucose levels. Waist circumference ≥90 cm for Chinese men and ≥80 cm for Chinese women was defined as central obesity according to the 2004 IDF definition of metabolic syndrome [15].

### 2.3. Statistical Analysis

A chi-square test for categorical variables and the general linear model (GLM) procedure for continuous variables were used to compare differences in prevalence and in age-adjusted means among different glucose categories. The linear association of CRP and GGT with plasma glucose and HbA1c was tested using linear regression model adjusting for age, school years, family history of diabetes, WC, LDL, TG, and SBP. Stepwise logistic regression analysis was performed to estimate the odds ratio (OR) and 95% confidence intervals (CI) for the prevalence of diabetes corresponding to a one standard deviation (SD) increase in continuous variables. Age, school years, alcohol-drinking, smoking, family history of diabetes, BMI, SBP, LDL, TG, TC, CRP and GGT were fitted in the multivariate model. For all analyses, variables with skewed distribution, such as CRP, and GGT, were log transformed before data analysis. All analyses were performed using SPSS (Version 15.0; SPSS Inc, Chicago, IL, USA). A *P*-value less than  .05 (two tailed) was considered statistically significant.

## 3. Results

 The baseline characteristics of the study population were summarized in Table 1. The respective prevalence of newly diagnosed diabetes by glucose or HbA1c criteria was 13.1% versus 10.5% in men and 11.4% versus 10.0% in women. Compared with individuals without diabetes by glucose criterion, those with newly diagnosed diabetes were older and more obese, hypertensive, dyslipidemia, and insulin resistant in men and in women. They also had significantly higher geometric mean of GGT and CRP. Family history of diabetes was more common in patients with diabetes than in those without it. Current smoking and alcohol-drinking made no difference between diabetic and non-diabetic groups of either gender. In comparison with subjects without diabetes by HbA1c criterion, those with newly diagnosed diabetes by HbA1c criterion had higher serum FPG, 2hPG, HDL, TC, and fasting insulin levels in men and higher WC, serum FPG, 2hPG, CRP, and fasting insulin levels in women. There was no difference in GGT concentrations between diabetes and non-diabetes defined by the HbA1c criterion. 

The correlation coefficients for CRP were statistically significant with either glucose levels or HbA1c levels in women but not in men, whereas those for GGT were significant with 2hPG in both men and women and with FPG in women only (Table 2). 

 The OR for newly diagnosed diabetes by the glucose criterion were significantly higher for GGT but not for CRP in men and women, which was, however, not observed for newly diagnosed diabetes by the HbA1c criterion (Table 3). But in the present study, an HbA1C level of 6.5% recommended by the International Expect Committee has a sensitivity and specificity of 33.5% and 87.8% among the participants. According to our previous report [16], the optimal HbA1C cutoff point for newly diagnosed diabetes in this study population was 5.6%, which was lower than the recommended value of 6.5%. By taking a cutoff of 5.6% with the sensitivity of 74.2% and specificity of 65.2%, the OR for newly diagnosed diabetes by the HbA1c criterion remained unsignificant for CRP and GGT in men and women. There was a significant interaction of the WC with the CRP (*P* = .039) and with the GGT (*P* < .001) in women, but not in men. A stratified analysis by the WC levels was, thus, performed to check whether the association between the CRP (or GGT) and diabetes was mediated through obesity. The results showed that the CRP was associated with the presence of diabetes only in obese women, not in women with low WC. The GGT was positively associated with diabetes in spite of the WC levels in both men and women (Table 3). In a subgroup of individuals with fasting insulin measures (sample size = 2125), the analysis was also made by fitting fasting insulin into the stepwise logistic model. After fitting with fasting insulin, the OR for newly diagnosed diabetes by the glucose criterion were significantly higher for GGT in men (OR = 1.33, 1.05–1.69) and in women (OR = 1.47, 1.18–1.72) but for CRP only in obese women (OR = 1.23, 1.04–1.56), which were similar with the results from the model without fasting insulin within the same subgroup of individuals. 

The prevalence of an FLI of ≥60 is 25.5% in men and 9.6% in women in our present study, and elevated serum GGT levels was associated with diabetes in both low (FLI <60) (OR = 1.02, 1.01–1.03) and high (FLI ≥ 60 ) (OR = 1.07, 1.02–1.18) FLI groups.

## 4. Conclusions

In this population-based cross-sectional Chinese study, we found that the elevated GGT concentrations were independently associated with the presence of diabetes defined by the glucose criterion in both men and women, while the effect of elevated CRP was significant only in obese women. Diabetes defined by the HbA1c criterion was associated with none of the two. 

Previous studies led to conflicting results as to whether there was an association of serum CRP levels with type 2 diabetes. Cross-sectional [8] and longitudinal studies [9] have shown a positive association between the CRP and diabetes in women but not in men. A hazard ratio of 7.60 (95% CI [4.43, 13.04]) for incidence of diabetes was reported in women in the MONICA/KORA Augsburg case-cohort study [9]. Another study in Hoorn in the Netherlands with only 140 men and 139 women revealed an independent association in men but not in women with incidence of diabetes [5]. In the Hisayama study in Japan, increased CRP level was a significant predictor of incident diabetes in both genders in the general Japanese population [7], and in the Hong Kong Cardiovascular Risk Factors Prevalence Study, CRP was found to independently predict the risk of progressing to diabetes in Chinese with IGT, but the data was not separately analyzed by genders due to the small sample size [6]. 

Recent studies demonstrated that elevated serum CRP levels were associated with obesity and insulin resistance [17, 18]. These studies suggested that the effect of the CRP on plasma glucose levels was mediated via obesity and insulin resistance. First, some studies have shown that reductions in CRP levels were strongly correlated with the amount of weight loss, suggesting a direct link between CRP and obesity [19]. Thus, obesity-triggered in*ﬂ*ammation could have a greater effect on the pathogenesis of type 2 diabetes. Second, some studies have shown that CRP, a sensitive marker of chronically subclinical inflammation, emerged to be part of the insulin resistance syndrome and an important factor in the pathogenesis of diabetes [17]. Several possible reasons might explain our finding that CRP was associated with diabetes only in obese women, and the main factors are presumably sex differences regarding body composition and sex hormone levels. First, the correlation between CRP and WC was stronger in women than in men (0.25 versus 0.11) in our study; moreover, women have a higher percentage of body fat than men [20], especially in obese women, so CRP may have a greater role in the development of insulin resistance and type 2 diabetes in women. Second, there may be an interaction between endogenous sex hormones and subclinical inflammation with the risk factors of diabetes [21, 22].

 Our observation that elevated GGT was an independently risk factor for diabetes by the glucose criterion in both men and women in this Chinese population is in line with results from some other studies [11, 12], which were performed in the general population. Several possible mechanisms may explain how GGT levels affect the development of diabetes. First, high GGT levels may indicate fat deposition in the liver which often causes hepatic insulin resistance, further systemic insulin resistance and hyperinsulinaemia [23, 24]. Thus, GGT could be a marker of the insulin resistance syndrome. Second, GGT plays an important role in the defensive response to oxidative stress. It is noteworthy that hyperglycemia, such as that shown by IGT/IFG, can induce the predominance of oxidative stress over antioxidative defense systems. The oxidative stress can contribute to more pronounced hyperglycemia, with this vicious cycle finally ending by inducing diabetes [25]. Third, inflammation may be one of the mechanisms, and one study showed that elevated serum GGT levels could be the expression of subclinical inflammation [26], which also leads to the pathogenesis of type 2 diabetes. 

In a cohort of Korean adults, fatty liver, as assessed by ultrasound, was predictive of diabetes independent of other risk factors [27], and a fatty liver index calculated based on levels of GGT, TG, BMI, and WC could predict the development of diabetes during a followup of a French cohort [28]. Elevation of the GGT has been considered to be a marker of the fatty liver and reported to precede the development of diabetes in a number of prospective studies [29, 30]. Our study showed that the association of GGT with type 2 diabetes was independent of the fatty liver index. However, whether the fatty liver index derived from a case-control Italian study can accurately assess the presence of the fatty liver in a Chinese population is not known and need to be further investigated. 

It is noteworthy that GGT, as a diabetes risk factor, is less important than a positive family history of diabetes and elevated waist circumference, which have been included in a simple Qingdao Diabetes Risk Score derived from the study population [31]. But GGT may be a useful marker to study the pathogenesis of diabetes. The study also revealed that the phenotypes of diabetes diagnosed by glucose criteria may differ from that by the HbA1c criteria, which requires further investigation. 

The study has several strengths. First, all diabetic subjects were newly diagnosed according to standard 2-hour OGTT criteria or HbA1c criteria; previously diagnosed diabetes were excluded to avoid the impact of the hypoglycaemic medications, modification in lifestyles, and the duration of diabetes which might reduce the weight of the patients. Second, this is a population-based study which is a representative of the Chinese population in general. The sample size is large enough to enable data analysis separately for men and women. 

 However, several limitations should be considered. First, since the current study was cross-sectional, it was not possible to establish the causality between elevated serum CRP/GGT and type 2 diabetes. Second, an ultrasoundgraphic scan to assess the fatty liver was not performed in the current study, and the possible association of the fatty liver with the presence of diabetes, and with the elevated GGT could not be accurately explored. The fatty liver index is only a surrogate measure of the fatty liver but not validated in a Chinese population yet. 

In summary, the effect of elevated CRP on diabetes defined by the glucose criteria was mediated through obesity, but elevated GGT was an independent risk factor for diabetes by the glucose criteria in this Chinese population. None of the two was, however, associated with the diabetes diagnosed by HbA1c criterion. The differences in the results between different criteria may reflect the fact that diabetic individuals defined by different criteria may have different phenotypic and prognostic characteristics. This is worthy to be further investigated with prospective studies. The contribution of obesity and fatty liver needs also to be further investigated.

##  Conflict of Interests

The authors declare that they have no conflict of interests.

## Figures and Tables

**Table 1:** Baseline characteristics of participants according to diabetes status defined according to either glucose or HbA1c criteria in individuals without a prior history of diabetes.

	FPG ≥7.0 mmol/L and/or 2hPG ≥11.1 mmol/L				HbA1c ≥6.5%			
	Men		Women		Men		Women	
	No	Yes	No	Yes	No	Yes	No	Yes
Number (%)	1014 (86.9)	153 (13.1)	1423 (88.6)	184 (11.4)	1044 (89.5)	123 (10.5)	1446 (90.0)	161 (10.0)
Age (years)	48.4	52.8	48.3	55.3	49.2	48.4	49.0	49.4
	(47.5, 49.3)	(52.0, 53.6)*	(48.0, 48.6)	(54.6, 56.0)*	(48.9, 49.5)	(47.5, 49.3)	(48.7, 49.3)	(48.5, 50.3)
School years>9 (%)	45.3	43.4	36.5	25.3*	46.4	30.9*	36.8	17.2*
Current smoking (yes, %)	47.1	47.7	1.3	2.2	46.4	52.0	1.5	0.7
Alcohol-drinking (yes, %)	48.8	49.0	1.6	0.5	48.5	51.2	1.6	0.7
Family history of diabetes (yes, %)	12.3	24.2*	16.4	22.8*	13.9	13.0	13.7	11.9
Occupational activity (%)								
Light	15.1	14.0	14.7	15.9	15.4	12.2	13.2	12.0
Moderate	46.4	52.7	61.8	64.0	48.0	41.5	45.4	47.0
Heavy	38.5	33.3	23.5	20.1	36.6	46.3	41.4	51.0
Body mass index (kg/m^2^)	25.6	26.1	25.6	27.0	25.7	25.9	25.8	26.1
	(25.4, 25.8)	(25.5, 26.7)*	(25.5, 25.8)	(26.5, 27.5)*	(25.5, 25.9)	(25.3, 26.5)	(25.6, 26.0)	(25.6, 26.7)
Waist circumference (cm)	87.1 (86.5, 87.7)	89.6 (88.0, 91.1)*	81.5 (81.0, 81.9)	86.4 (84.9, 87.8)*	87.4 (96.8, 87.9)	87.7 (85.8, 89.5)	81.8 (81.4, 82.3)	85.2 (83.5, 86.8)*
Systolic blood pressure (mmHg)	132	140	129	138	133	134	131	131
	(131, 133)	(137, 143)*	(128,130)	(135, 141)*	(132,134)	(132,136)	(131, 132)	(128, 134)
Diastolic blood pressure (mmHg)	87 (86, 88)	90 (88, 92)*	83 (82,84)	87 (86, 88)*	87 (86, 88)	89 (86, 91)	84 (83, 85)	84 (82, 86)
Fasting plasma glucose (mmol/L)	5.33 (5.29, 5.38)	8.00 (7.55, 8.46)*	5.37 (5.34, 5.42)	7.66 (7.25, 8.08)*	5.59 (5.53, 5.65)	6.44 (6.11, 6.77)*	5.58 (5.53, 5.63)	6.13 (5.90, 6.36)*
2 hours post-load plasma glucose (mmol/L)	6.23 (6.14, 6.33)	13.2 (12.4, 14.1)*	6.63 (6.55, 6.71)	13.20 (12.44,13.96)*	6.77 (6.62, 6.91)	9.76 (8.69, 10.83)*	7.12 (6.99, 7.24)	9.78 (8.81, 10.75)*
Fasting insulin (*ρ* mol/L)*||*	30.72	37.15	39.98	49.66	28.11	32,47	37.41	42.07
	(29.67, 31.77)	(35.45, 38.85)*	(38.85, 41.11)	(48.36, 50.96)*	(25.68, 30.54)	(31,16, 33.78)*	(36.14, 38.68)	(40.18, 43.06)*
Low density lipoprotein cholesterol (mmol/L)	2.96	3.20	2.98	3.37	2.97	3.12	3.04	2.94
	(2.90, 3.01)	(3.03, 3.36)*	(2.93, 3.03)	(3.22, 3.52)*	(2.91, 3.03)	(2.94, 3.30)	(2.99, 3.08)	(2.78, 3.11)
High density lipoprotein cholesterol (mmol/L)	1.62	1.61	1.67	1.59	1.61	1.72	1.66	1.71
	(1.60, 1.65)	(1.54, 1.69)	(1.65,1.69)	(1.52, 1.66)*	(1.58,1.64)	(1.65, 1.80)*	(1.64,1.68)	(1.64,1.76)
Triglycerides (mmol/L)	1.37	1.49	1.15	1.48	1.45	1.42	1.22	1.24
	(1.33, 1.42)	(1.34,1.64)*	(1.11, 1.18)	(1.32,1.60)*	(1.40,1.51)	(1.19, 1.65)	(1.17,1.26)	(1.03, 1.45)
Total cholesterol (mmol/L)	5.22	5.63	5.17	5.73	5.24	5.43	5.25	5.15
	(5.16, 5.28)	(5.46, 5.81)*	(5.12, 5.22)	(5.57, 5.89)*	(5.18, 5.31)	(5.23, 5.63)*	(5.19, 5.29)	(4.97, 5.34)
C-reactive protein (mg/dl)§	0.87	1.26	0.72	1.26	0.91	0.94	0.76	0.90
	(0.76, 0.98)	(1.08, 1.44)*	(0.66, 0.78)	(1.06,1.46)*	(0.78,1.04)	(0.60, 11.14)	(0.70, 0.82)	(0.88, 0.92)*
Alanine aminotransferase (U/L)	13.1	16.9	11.0	13.1	13.7	13.5	11.2	11.7
	(12.4, 13.9)	(14.8, 18.9)*	(10.4, 11.6)	(11.2, 14.9)*	(12.9,14.4)	(11.2, 15.8)	(10.5, 11.7)	(9.6, 13.7)
Gamma-glutamyltransferase (U/L)§	28.6	41.4	15.5	23.6	29.9	30.1	16.2	17.4
	(26.5, 30.7)	(37.2, 45.6)*	(15.2, 15.8)	(22.0, 25.2)*	(27.8, 32.0)	(27.3, 32.9)	(15.8, 16.6)	(16.0, 18.8)

Data are age-adjusted mean (95% confidence interval) or percentages as indicated. *P < .05, diabetes versus nondiabetes by the same criteria within the same gender. §Geometric mean (95% CI). *||*With missing data *n* = 2125.

**Table 2:** Standard *β* coefficients for C-reactive protein (CRP) and gamma-glutamyltransferase (GGT) in association with fasting, 2 hours plasma glucose concentrations (mmol/L) and HbA1c (%) in individuals without a prior history of diabetes.

	Fasting plasma glucose	2 hours plasma glucose	HbA1c
Men (*n* = 1167)			
CRP (mg/dL)	0.03	0.04	0.03
GGT **(**U/L)	0.02	0.11*	0.03
Women (*n* = 1607)			
CRP (mg/dL)	0.08*	0.10^†^	0.06*
GGT **(**U/L)	0.15^†^	0.12^†^	0.04

Adjusted for age, school years, family history of diabetes, waist circumference, systolic blood pressure, triglycerides, and low density lipoprotein cholesterol. CRP and GGT are logarithmic transformed. *P < .01, ^†^ *P* < .001.

**Table 3:** Odds ratio (95% CI) for newly diagnosed diabetes corresponding to a one-standard deviation increase in continuous variables stratified by waist circumference categories. Only the variables entered into the final logistic regression model are presented.

	Standard deviation	FPG ≥7.0 mmol/L and/or 2hPG ≥11.1 mmol/L OR (95% CI)	HbA1c ≥6.5% OR (95% CI)	HbA1c ≥5.6%* OR (95% CI)
*Men*				

*Waist circumference *<90* (cm) ( * *n* = 671)				

Age (year)	10.41	1.46 (1.12,1.89)	—	—
Positive family history of diabetes	—	3.04 (1.60,5.77)	—	—
Gamma-glutamyltransferase (U/L)	0.72	1.59 (1.29,1.96)	—	—
Systolic blood pressure (mmHg)	19.25	1.38 (1.08,1.76)	—	1.30 (1.10,1.53)
Total cholesterol (mmol/L)	1.03	—	1.32(1.04,1.66)	—
School years >9 (≤9)	—	—	0.59(0.34,0.98)	—

*Waist circumference *≥90* (cm) ( * *n* = 496)				

Age (year)	10.41	1.62 (1.21,2.19)	—	—
Positive family history of diabetes	—	2.42 (1.28,4.58)	—	—
Low-density lipoprotein cholesterol (mmol/L)	0.97	1.61 (1.23,2.10)	—	1.61 (1.28,2.02)
Triglyceride (mmol/L)	1.10	1.64 (1.26,2.14)	—	—
School years >9 (≤9)	—	—	0.46 (0.25,0.85)	—
Body mass index (kg/m^2^)	2.52	—	1.50 (1.05,2.15)	1.27 (1.01,1.60)
Smoking	—	—	2.29 (1.24,4.22)	—

*All (* *n* = 1167)				

Age (year)	10.41	1.65 (1.37,1.99)	—	—
Positive family history of diabetes	—	2.35 (1.50,3.66)	—	—
Gamma-glutamyltransferase (U/L)	0.72	1.42 (1.18,1.72)	—	—
Low-density lipoprotein cholesterol (mmol/L)	0.97	1.26 (1.06,1.49)	—	1.32 (1.17,1.49)
Triglyceride (mmol/L)	1.10	1.30 (1.11,1.52)	—	—
School years >9 (≤9)	—	—	0.50 (0.34,0.74)	—
Total cholesterol (mmol/L)	1.03	—	1.24 (1.04,1.47)	—

*Women*				

*Waist circumference *<80* (cm) ( * *n* = 667)				

Age (year)	9.74	1.75 (1.26, 2.44)	—	—
Positive family history of diabetes	—	2.42 (1.12,5.21)	—	—
Gamma-glutamyltransferase (U/L)	0.54	1.83 (1.32,2.54)	—	—
School years >9 (versus ≤9)	—	—	0.31 (0.15,0.64)	—
Systolic blood pressure (mmHg)	22.42	—	—	1.34 (1.09,1.64)

*Waist circumference *≥80* (cm) ( * *n* = 940)				

Age (year)	9.74	1.47 (1.19,1.82)	—	1.19 (1.04,1.36)
C-reactive protein	1.02	1.30 (1.11,1.51)	—	—
Gamma-glutamyltransferase (U/L)	0.54	1.56 (1.27,1.91)	—	—
Systolic blood pressure (mmHg)	22.42	1.22 (1.02,1.48)	—	—
Triglyceride (mmol/L)	0.80	1.57 (1.30,1.89)	—	—
School years >9 (versus ≤9)	—	—	0.44 (0.26,0.75)	—

*All (* *n* = 1607)				

Age (year)	9.74	1.44 (1.19,1.73)	—	1.15 (1.02,1.28)
Positive family history of diabetes	—	1.66 (1.09,2.51)	—	—
Gamma-glutamyltransferase (U/L)	0.54	1.57 (1.31,1.87)	—	—
C-reactive protein (mg/dl)	1.02	1.21 (1.00,1.45)	—	—
Low-density lipoprotein cholesterol (mmol/L)	0.92	1.22 (1.04,1.43)	—	1.16 (1.04,1.29)
Triglyceride (mmol/L)	0.80	1.48 (1.26,1.74)	—	—
Systolic blood pressure (mmHg)	22.42	1.26 (1.06,1.49)	—	1.30 (1.10,1.53)
School years >9 (≤9)	—	—	0.36 (0.23,0.55)	—

Age, body mass index, gamma-glutamyltransferase, low-density lipoprotein cholesterol, triglyceride, total cholesterol, C-reactive protein, systolic blood pressure, family history of diabetes, school years, smoking, and alcohol-drinking are fitted in the models.C-reactive protein and gamma-glutamyltransferase were logarithmic transformed. *Optimal cutoff value for diagnosis of diabetes in this study population [16].
